# Disclosing the person in renal care coordination: why unpredictability, uncertainty, and irreversibility are inherent in person-centred care

**DOI:** 10.1007/s11019-022-10113-z

**Published:** 2022-09-20

**Authors:** Martin Gunnarson

**Affiliations:** Centre for Studies in Practical Knowledge, School of Culture and Learning, Södertörn University, Huddinge, Sweden

**Keywords:** Person-centred care, Renal care coordinator, Hannah Arendt, Speech, Action, Uncertainty

## Abstract

This article explores an example of person-centred care: the work of so-called renal care coordinators. The empirical basis of the article consists of qualitative interviews with renal care coordinators, alongside participant observations of their patient interactions. During the analyses of the empirical material, I found that that one of the coordinators’ most fundamental ambitions is to get to know *who* the patient is. This is also a central tenet of person-centred care. The aim of the article is not only to argue for the plausibility of this tenet, but also, and more importantly, to highlight and explore its implications in the context of healthcare, through the example of renal care coordination. By drawing on the philosophy of Hannah Arendt, the article shows that the disclosure of *who* the patient is that takes place in person-centred care requires speech and action, which are modes of human activity that initiate processes characterized by unpredictability, uncertainty, and irreversibility. This unpredictability, uncertainty, and irreversibility, found to be inherent in person-centred care, is then discussed in relation to the pursuit of certainty characterizing contemporary evidence-based medicine. At the end of the article the conclusion is drawn that, if healthcare is to be person-centred, it must find ways of accommodating the contradictory pursuits of certainty and uncertainty found in evidence-based medicine and person-centred care respectively.

## Introduction

Ever since the mid twentieth century a struggle has been waged against the dehumanising, objectifying, and paternalistic tendencies of modern medicine (see e.g. May and Mead 1999; Gunnarson 2016, 180–185). Although the goal of this struggle has been conceptualized in different ways—as “holistic care” and “patient-centred care” for example—its main tenet has remained the same: when caring for a patient, medical practitioners must see and treat this patient as a whole person, not as a physiological entity affected by a discrete disease. Lately, this goal has been conceptualised in terms of “person-centred care,” emphasising even more that medical action should be centred around and adapted to the meaningful lifeworld of a specific person.

In this article I explore an example of such person-centred care: the work of so-called renal care coordinators. The empirical basis of the article consists of qualitative interviews with renal care coordinators, alongside participant observations of their patient interactions. During my analyses of the empirical material, I realised that one of the coordinators’ most fundamental ambitions is to get to know *who* the patient is. This is also a central tenet of person-centred care (see e.g. Ekman et al. 2011, 249; Kristersson Uggla 2022, 6). The aim of the article is not only to argue for the plausibility of this tenet, but also, and more importantly, to highlight and explore its implications in the context of healthcare, through the example of renal care coordination. By turning to the philosophy of Hannah Arendt, which is yet to be used in research on person-centred care, my ambition is to contribute to a deeper understanding of the implications of this form of care.

## Previous research on person-centred care

One of the first calls for a care centred around the person came from social psychologist Tom Kitwood. In his seminal work *Dementia Reconsidered: The Patient Comes First*, published for the first time in 1997, he argued that the narrow understanding of dementia as “an organic brain disease,” which had dominated care approaches until then, and the cultural view that dementia entails “a loss of personhood” had led to a depersonalisation of people with dementia, excluding them from “the world of persons” (Kitwood 2019, 26, 32, 39). This amounted to an encroachment of the inviolable value of the person, Kitwood contended. What was needed, he argued, was a care that acknowledges and puts the person first (Kitwood 2019, 27).

In the book, Kitwood offers a definition of personhood that has been highly influential in the further theorization of person-centred care, especially in the context of dementia. Personhood, he states, “is a standing or status that is bestowed upon one human being, by others, in the context of relationship and social being” (Kitwood 2019, 27). For Kitwood, then, personhood is fundamentally relational. It is by being recognised by another as a person that we become a person. Here Kitwood is inspired by Martin Buber and his writings on the I-Thou relation. In Kitwood’s interpretation, personhood results from an authentic I-Thou meeting since it involves an openness towards, a true presence with and an awareness of a unique other (Kitwood 2019, 30). According to this understanding, to ask *who* the patient is—a central tenet of person-centred care—entails engaging in an I-Thou relationship with him or her.^1^

In the wake of Kitwood’s book, the concept of person-centred care has gained enormous traction within the caring sciences. Numerous scholars have developed and criticised Kitwood’s conceptualisation of personhood and person-centred care, in the context of dementia and elsewhere (see e.g. Dewing 2008; Chenoweth et al. 2009; McCormack and McCance 2010; Buetow 2016; Naldemirci et al. 2018). Some have argued that it should be reconceptualised as relationship-centred care, emphasising the relational aspect so essential for Kitwood (Beach et al. 2005). Others have launched the concept of “person-centred fundamental care,” which adds several dimensions to Kitwood’s original model with the aim to capture the “complexity and multidimensionality” of caring practices (Kitson 2018, 100). While the relational nature of care is at the centre of fundamental care, it also directs attention to the psychosocial and physical needs of each patient, and the practical tasks necessary for fulfilling these, as well as the contextual dimensions into which the needs and tasks are embedded, such as cultural, regulatory, and institutional dimensions (Kitson 2018, 101).

In a recent text, written from the perspective of person-centred fundamental care in the context of dementia, Tieu et al. (2022) criticise Kitwood’s notion of personhood. Since it hinges on the bestowment of the status of personhood of one upon another, they contend, this notion is more an “ethical imperative”—stating that one *should* bestow the other with personhood—than a “rigorous philosophical definition” (Tieu et al. 2022, 7). As an alternative—one that they argue should permeate all notions of person-centred care—they propose to ground the concept of personhood in the concept of self. As human beings we experience “a sense of ourselves” or “a sense of identity,” which “entails the integration of the social, relational and biographical dimensions of personhood,” they write (Tieu et al. 2022, 8). Furthermore, they contend, a person’s sense of identity, and the preservation thereof, is integral to her or his autonomy and empowerment, two concepts that have been essential to the theorisation of person-centred care. The form of autonomy that Tieu et al. articulate is a form of relational autonomy, relying on the nature of the relationships that a person has with others (cf. Folkmarsson Käll and Zeiler 2014). Now, several layers of meaning have been added to the question “who are you?” in the context of healthcare. Not only does it entail engaging in an I-Thou relationship with the other, but it also involves inquiring into her or his biography, social standing, and fundamental needs and wishes, as well as engaging in actions that promote and preserve her or his autonomy and empowerment.

Another influential source of theoretical inspiration for research on person-centred care comes from the French philosopher Paul Ricoeur.^2^ In a recent article Bengt Kristensson Uggla (2022) argues that the “triadic structure” of Ricoeur’s theory of the person—in which personhood is rooted in all three personal pronouns—“reveals a composite communicative structure which relates *self-understanding* (of a ‘me’ in the first person) to *dialogical understanding* (of a ‘you’ of second person), as well as to *objectifying explanations* (in terms of a ‘him/her/it’ in third person)” (Kristensson Uggla 2022, 4). This structure, he argues, provides the ground for an ethical approach that can aid the endeavour of person-centred care since it establishes a relationship between the “inner truths” emanating from the first person and “outer truths” emerging from the third person (Kristensson Uggla 2022, 6). Rather than providing universal ethical principles, this approach is a “little ethics” that is cultivated in and through practice and that cannot be fulfilled without an orientation towards the unique personhood of the involved actors, Kristensson Uggla contends (Kristensson Uggla 2022, 4, 5).^3^ Asking *who* the patient is from this perspective entails seeing her or him as a unique experiencing being embedded in relational practices and institutional contexts in and through which she or he acts and is acted upon.

From the theoretical approaches to person-centred care described above, one can get a sense of how it can be distinguished from patient-centred care. While the latter, in the words of Lambert et al. (1997, 31), largely remains “a model of biomedical care” focused on the *role* of the patient—the *what*—the former is understood as a form of care that extends beyond the realm of medicine towards the lifeworld of the person—the *who* (see also Håkansson Eklund et al. 2019).

Despite this, the desire to retain person-centred care within a biomedical frame is considerable. An indication of this is the plethora of scientific publications that offer standardised models for implementing it and make use of biomedical methods for studying and evaluating it (see e.g. Chenoweth et al 2009; Olsson et al. 2016; Fors et al. 2016; Hansson et al. 2017). In their influential paper published in 2011, for example, Ekman et al. advance a three-step model,^4^ which has subsequently been widely used and turned into a “PCC philosophy” termed “Gothenburg person-centred care (gPCC)” (Hansson et al. 2017, 2). In several studies, this PCC philosophy is used as a basis for testing and evaluating the efficacy of person-centred care, not infrequently by means of randomised control trials, calculating its effects on measurables such as “length of hospital stay” (LOS), “activities of daily life” (ADL), “health-related quality of life” (HRQoL) and “General Self-efficacy” (GSES) (Ekman et al. 2012; Olsson et al. 2016; Hansson et al. 2017). The present article questions the possibility of implementing person-centred care by means of such models and the suitability of evaluating it by means of quantitative and quantifiable measurables such as these.

As this summary of some of the main directions in the research on person-centred care has shown, several implications of asking *who* the patient is in person-centred care have already been observed. However, as I aim to show here, there are central implications still missing from the literature. In what follows, I will use Hannah Arendt’s concept of action to provide an empirically grounded analysis that shows the inherence of unpredictability, uncertainty, and irreversibility in person-centred care.

## Materials and methods

This article is based on empirical material consisting of in-depth interviews with renal care coordinators and participant observations of their work. The material was collected as part of a sub-study of a broader research project on existential issues and the role of existential philosophy in healthcare.^5^ The sub-study focused on the ways in which existential issues emerge and are handled by so-called “care coordinators.” My aim was to explore two forms of care coordination: the work of care coordinators in renal care and the work of contact nurses in cancer care. However, work had barely begun on the second group when the pandemic broke out. Consequently, I was unable to collect sufficient material about them. For this reason, I have had to limit my study to the renal care coordinators’ practices, on which I conducted participant observations from the fall of 2018 to the two first months of 2020. In June 2021, I met three of the four renal care coordinators included in the first part of the study for individual, in-depth interviews.^6^

My interest in the existential dimensions of coordinators’ practices already served to ground my methodological approach. In order to understand how existential issues emerge and are dealt with in care coordination, I needed to get close to the practice, which participant observation allowed me to do. But I was also interested in the professionals’ reflections on these dimensions of their practice. Hence the decision to conduct in-depth interviews. Moreover, due both to the potential sensitivity of existential issues and the possible unease of being observed while working, I limited the number of renal care coordinators in the study to four. This way, I was able to get to know the participants somewhat and build a trustful relationship with them.

A term that captures, more specifically, the type of participant observation that I conducted is “shadowing,” as conceptualised by Barbara Czarniawska (Czarniawska 2007; 2014). In shadowing, emphasis is placed on observation rather than participation. As a researcher, one follows, or “shadows,” one’s key research participants as they engage in their practices. During my observations, I did not actively participate in the renal care coordinators’ work, but followed them to and from their meetings with patients, hung out with them before, after and between meetings, and observed some of their interactions with colleagues.^7^ This illustrates the mobility of shadowing (McDonald and Simpson 2014). It enables the researcher to get close to the studied practice, by following it around, while still remaining somewhat distant. The advantage of this, in Czarniawska’s view, is that it promotes reflection; it allows the research participants, but also to some extent the researcher, to view themselves through the eyes of another (Czarniawska 2007, 56). This was apparent during my fieldwork. Not only did the renal care coordinators tell me that my presence made them reflect on their practice, but they also engaged in extensive reflections with me, about both their work and my study.

During my shadowing, I was clearly an outsider. Unlike some hospital ethnographers, I did not attempt to blend in by playing the role of a medical professional and putting on a white coat (van der Geest and Finkler 2004). Rather, I tried to be as explicit as possible about my role as a non-medical researcher and the purpose of my presence, towards all the participants, renal care coordinators as well as patients. As I argued in the previous paragraph, the relative detachment from the practice that this creates has several epistemological benefits. However, my candidness about my role and aim was also ethically motivated. In order for the renal care coordinators and patients to make an informed decision about their participation in the study, they had to know who I was and why I was there.

When I conducted my last observation early in 2020, I had observed a total of 21 conversations between patients and renal care coordinators, the majority of which I audio recorded and transcribed verbatim.^8^

The interviews I conducted at the end of my fieldwork were to a great degree informed by my experiences of shadowing the renal care coordinators. By that point, I was familiar with their practice, as they were with me and my study. Still, I wanted to know more about their perspectives on their role and practice and their thoughts and feelings about the existential issues I was interested in. The interviews thus allowed them to give extended, first-person accounts that included these dimensions (Holstein and Gubrium 1995). The interviews were semi-structured in the sense that they revolved around a number of questions I had prepared beforehand, while at the same time were open to the direction the respondents wanted to take (Davies 2008, 105–106).^9^ I audio recorded the interviews and transcribed them verbatim.

During and after the fieldwork I read all the transcripts and my fieldnotes multiple times, coding the material and ordering the codes into themes based on the main research questions of my sub-project and theoretical perspectives rooted in existential philosophy (Braun and Clarke 2006). Thus, the thematic analysis was, from the outset, informed by the philosophical literature I read (Davies 2008).^10^ It was out of this analytic process that the theme of this article emerged, at which point I deepened my exploration of the material concerning this theme and the philosophical literature together with which it emerged. What struck me was that the renal care coordinators always try to get to know *who* the patient is. This ambition is ubiquitous. With the help of Hannah Arendt’s philosophy, I saw that this requires a particular form of human activity: *action*, in Arendt’s sense of the term. What also stood out in the empirical material was the *unpredictability*, *uncertainty*, and *irreversibility* that Arendt associate with action. As I will argue below when I develop Arendt’s concepts, the disclosure of *who* the patient is, and the implications thereof rely on words uttered and deeds performed that cannot be predicted in advance and whose consequences are unpredictable, since they are performed by and directed to unique persons. To gain an understanding of this disclosure, therefore, one must get a sense of the situational circumstances orienting it. This means that the ways in which and the degree to which renal care coordinators get to know the patients they meet vary. Rather than giving an account of this variation, I conduct an in-depth analysis of a single conversation between a coordinator (Pia) and a patient (Fredrik) in this article. This is so because I want to give a concrete account of how the particular persons involved and the circumstances under which they meet influence the ways in which the question “who are you?” is posed and answered, and the implications thereof. The interviews with the coordinators provide a context to this single conversation, showing the ubiquity of the ambition to get to know who the patient is and the fact that this ambition is shaped by the particularities characterising each care relation.

Before my fieldwork started, the study was approved by the Swedish Research Ethics Board (*Regionala Etikprövningsnämnden*).^11^ I began by contacting the renal care units at two hospitals and was immediately able to make contact with care coordinators willing to participate in the study. Their participation was subsequently approved by their heads of unit. Two of the four renal care coordinators then helped me recruit patients for the study. They sent out the information letter directed to patients that I had formulated as part of my application to the research ethics board, and also asked the patients verbally, either by phone or in person, whether they were willing to participate. The patients then either brought the signed informed consent form, attached to the information letter, to the meeting I was scheduled to observe or signed the form while at the meeting.

During the study I continuously discussed the selection of patients with the renal care coordinators. My aim was to get a sense of how renal care coordination changes as the disease progresses and as the patient and coordinator became more familiar with one another. I therefore wanted to include patients who had no, moderate, or extended experiences of interacting with a renal care coordinator. The majority of patients included in the study are men, above the age of 50. This reflects the demographics of the disease. The participants mentioned in this article have for the sake of anonymity been pseudonymised.

Although I emphasise and explore the personhood of patients in this text, I still frequently use the word patient to the denote the person with whom the renal care coordinator interacts. The main reason for this is clarity. If I were to only use the word person, it would be more difficult for the reader to understand whom I am referring to and to separate this person from the renal care coordinator, who is also a person.

## The renal care coordinator

What, then, is a renal care coordinator? The role emerged out of an experienced need to support and educate patients who are in the so called “predialytic phase,” that is, persons who have been diagnosed with chronic kidney disease but who are not yet in need of life-sustaining treatments in the form dialysis or kidney transplantation. The renal care coordinator is but one way of meeting this need. According to American and European guidelines, patients should, at minimum, be assigned an “educator” who meets with the patient on several occasions and provides clear and unbiased information about the available treatment options (Goovaerts et al. 2015). Predialytic patients may also receive support and education by means of so called “multidisciplinary care” where practitioners from a range of professions—for example physicians, nurses, physiotherapists, dieticians and occupational therapists—meet at regular intervals to discuss the care provided to a particular patient (Strand and Parker 2012). The aim of this approach is to gain an overall picture of the patient’s situation in order to assist her or him in making beneficial lifestyle and treatment choices. Sometimes, but not always, such a multidisciplinary care team is led by a care coordinator or case manager, the role of whom is often to simultaneously coordinate the work of the multidisciplinary team and function as the patient’s main contact person.

In Sweden, where my research was conducted, the renal care coordinator is the most central actor for providing support and education to the predialytic patient. The extent to which there is a functioning multidisciplinary care team for her or him to lead varies between regions and hospitals (Njurförbundet 2020). However, according to the written job descriptions of the four renal care coordinators participating in this study, one of the main tasks of the profession is to coordinate and act as a contact person for the team around a single patient. Two other central tasks listed are to provide information and education tailored to specific patients’ needs—in order to increase patients’ knowledge about their disease state and their ability to engage in various forms of self-care—and to have recurrent conversations with patients—in order to increase patients’ participation in care actions and treatment choices, contribute to a feeling of security among them, and motivate them to choose some form of self-care dialysis. These job descriptions quite clearly convey what struck me as one of the most essential aspects of the renal care coordinators’ work when I first came into contact with them: to engage in continuous conversations with patients. This aspect of their work will be my focus in this article.

In the Swedish context, renal care coordinators are trained nurses working at the outpatient renal clinic. Here, patients who have been diagnosed with chronic kidney disease come for regular check-ups with a doctor. Chronic kidney disease is a progressive disease divided into five stages. It is typically when the disease has reached its fourth stage that patients are referred by their treating doctor to a renal care coordinator. Even though patients have lost about 70 percent of their kidney function at this stage, it can take several years before renal replacement therapies in the form of either dialysis or kidney transplantation are needed. Moreover, many patients do not experience any symptoms of the disease at this stage. It is therefore often referred to as a “silent disease.”

The frequency and content of the renal care coordinators’ meetings with patients vary due to several factors: the patient’s own needs and wishes; the speed of the progression of the disease; whether or not an important treatment decision has to be made; or if the patient has to be prepared, both physically and mentally, for the initiation of renal replacement therapies. Sometimes a year passes between meetings, in other cases only a couple of months. The meetings are rarely shorter than 30 min and may extend beyond an hour. The main component of these meetings is a conversation that takes place between the renal care coordinator and patient. As we shall see, these conversations may touch on a huge variety of topics.

The renal care coordinators do not themselves use the term person-centred care to describe their work. This is my interpretation. My ambition below is to demonstrate the plausibility of this interpretation through the empirical examples I give and my analyses of them.

## The revelatory power of speech and action

In her seminal work *The Human Condition* Hannah Arendt explores human activity, *vita activa*. Throughout the history of philosophy, she argues, *vita activa* has either been subordinated to contemplation – *vita contemplative*—or erroneously understood as lacking internal distinction (Arendt [1958] 2018, 17). Arendt wants to remedy these shortcomings. She begins by distinguishing between “three fundamental human activities: labor, work, and action,” which together constitute *vita activa* and which each correspond to “one of the basic conditions under which life on earth has been given to man” (Arendt [1958] 2018, 7). The condition to which labour corresponds is “life itself.” Labour is the activity by means of which we satisfy our most basic needs of survival, according to Arendt. Work, on the other hand, corresponds to the basic human condition of “worldliness.” Through the activity of work we create “an ‘artificial’ world of things,” she writes, a world in which we become individuals and which will outlive us. Action, finally, corresponds to the human condition of “plurality.” Human existence is characterised by plurality because “men, not Man, live on the earth and inhabit the world” (Arendt [1958] 2018, 7). Action is both rooted in and what actualises plurality. What makes us capable of action, according to Arendt, is the fact that we were born into the world as “newcomers,” as someone who was not there before. This “natality” is what enables us to “take an initiative, to begin […], to set something new in motion,” that is, to act (Arendt [1958] 2018, 177). Action, then, is to initiate something new, which is only possible because we are someone new. In action the act and the actor are inseparable.

As much as an act begins something new, it must answer the question “Who are you?”, according to Arendt (Arendt [1958] 2018, 178). Consequently, action is dependent on speech, which is itself a form action. Arendt writes:

[The] disclosure of who somebody is, is implicit in both his words and his deeds; yet obviously the affinity between speech and revelation is much closer than that between action and revelation, just as the affinity between action and beginning is closer than that between speech and beginning, although many, and even most acts, are performed in the manner of speech. (Arendt [1958] 2018, 178)

Action would not be action were it not accompanied by speech acts, since the actor would not be sufficiently revealed. At the same time, speech is itself a form of action, fundamentally rooted in the human condition of natality, without which there would not be a unique actor to reveal (cf. Loidolt 2017, 197). This does not mean that the words and deeds performed and uttered by someone must explicitly concern her or him. What matters is that they are of interest to the actors and speakers involved, “in the word’s most literal significance, something which *inter-est*, which lies between people and therefore can relate and bind them together.” Thus, even if “most words and deeds are *about* some worldly objective reality,” they are also “a disclosure of the acting and speaking agent,” Arendt continues (Arendt [1958] 2018, 182).

Arendt further claims that speech and other forms of action are characterised by unpredictability and uncertainty. This can be attributed to their natality, that is, their capability of setting something new in motion and inserting someone new into the world. For Arendt, unpredictability and uncertainty are inherent features of the new. She writes: “The fact that man is capable of action means that the unexpected can be expected from him, that he is able to perform what is infinitely improbable” (Arendt [1958] 2018, 178). This also applies to its consequences. When something new is initiated, how it will affect the already existing world is fundamentally unpredictable and uncertain. This applies both to the some*thing* that is initiated and the some*one* who is disclosed. Moreover, the person that is revealed and the process that is set in motion can never be fully known by the actors involved (Arendt [1958] 2018, 192). Action is a process, Arendt contends, the durability of which often extends beyond that of the things produced by work, and the strength of which “is never exhausted in a single deed but, on the contrary, can grow while its consequences multiply” (Arendt [1958] 2018, 233). In contrast to the objects we fabricate by means of work, which can be destroyed or will eventually perish, “action has no end,” she writes (Arendt [1958] 2018, 233). Speech and other forms of action, then, are not only unpredictable; they are also irreversible. In Arendt’s view, this is both a blessing and a curse. While our ability to begin something new is what liberates us, and the action processes we initiate may outlive us and afford us a certain immortality, the irreversibility, unpredictability, and uncertainty of action immediately strips us of any possibility to undo or fully control and understand what we have begun (Arendt [1958] 2018, 233–235). As we shall see, this tension, inherent in speech and other forms of action, is highly evident in the person-centred practice of renal care coordination.

As I mentioned above, for Arendt, action is the human activity that corresponds to the condition of plurality. As such, it is the only activity that goes on “directly between men” and that is “entirely dependent upon the constant presence of others” (Arendt [1958] 2018, 7, 23). Through speech and other forms of action, human beings direct themselves towards each other and thereby create and uphold a “realm of human affairs” (Arendt [1958] 2018, 183). This realm consists of a “space of appearance,” in which unique persons may appear for each other, and a “web of human relationships,” through which these persons may engage with one another (Arendt [1958] 2018, 184, 198). As such, action is fundamentally political. Without it, Arendt contends, the formal and institutional organisation of a political realm would not be possible. Consequently, when we engage in speech and other forms of action, our appearance as someone and our initiation of something new “always fall into an already existing web where their immediate consequences can be felt” (Arendt [1958] 2018, 184). This is another source of their irreversibility and uncertainty: due to their embeddedness in an already existing plural public realm, the processes they initiate can neither be undone nor fully controlled. Thus, speaking and acting means taking a risk. It entails throwing oneself into uncertainty, fully aware of the impossibility of controlling the implications of what one has begun and who one has become. Even though we quickly lose control of the consequences of our actions, Arendt states that our capacity to act is what sets us free (Arendt [1958] 2018, 235). “To *be* free and to act are the same,” she writes, indicating with the italicisation of the word “*be*” that we *are* not simply free, but that freedom is performative (Arendt 1960, 33). It is furthermore relational. Just as action, freedom requires “the company of other men” and a “common public space,” she writes (Arendt 1960, 30). Thus, we cannot be free without others; our capacity, in an act of freedom, to begin something new hinges on the presence of a web of relationships. Here, there are affinities with the relational autonomy Tieu et al. (2022) advance as a fundamental aspect of their concept of person-centred fundamental care.

Based on the above, one can argue that Arendt too offers a theory of personhood. It is when we act—primarily in the form speech—in a world together with others that we emerge as unique persons. Arendt writes:

In acting and speaking, men show who they are, reveal actively their unique personal identities and thus make their appearance in the human world […]. This disclosure of ‘who’ in contradistinction to ‘what’ somebody is – his qualities, gifts, talents, and shortcomings – is implicit in everything somebody says and does. (Arendt [1958] 2018, 179)

As this quote indicates, for Arendt, our personhood does not only reside within the public realm. Rather, our unique personal identity *appears* in it, as she writes, which suggests that there is already someone there who can appear. However, as Sofie Loidolt (2020) proposes, the private realm—in which we become persons through our love relationships and our development of a personal taste—and the public realm are always interconnected and mutually dependent. Moreover, she argues, Arendt has a phenomenological understanding of appearance that affords it a fundamentally performative character (Loidolt 2020, 178). Consequently, the *who* that appears in the public realm, through speech or any other form of action, cannot be separated from the *how* of this appearance, which in turn affects the *who* of the private realm. Here, the relational character of Arendt’s theory of personhood comes to light. According to Loidolt, Arendt “makes explicit that ‘performance’ means that the self appears to others on a stage and needs others to articulate and become itself” (Loidolt 2020, 178). Thus, for Arendt, personhood is not the result of the bestowment of the status of personhood of one upon another, as it is for Kitwood. Rather, it is the result of the appearance—read performance—of some*one* by means of speech and action, in and through the web of relationships that constitute the world. In Arendt’s view, then, asking *who* the patient is entails meeting her or him in a common world by engaging in speech and other forms of action that open up a space of appearance in which the patient may reveal who she or he is by speaking and acting.

However, in Arendt’s analysis there is a leaning towards action in the form speech when it comes to the disclosure of *who* someone is. In her words, the “affinity” between speech and revelation is greater than that between action and revelation (Arendt [1958] 2018, 178). As the case I study here—renal care coordination—is also primarily characterised by action in the form of speech, on account of the extended conversations patients and renal care coordinators have with each other, there is a risk that other forms of care, where this type of verbal interaction might not be possible, are excluded from the analysis. Persons with advanced dementia, for example, might be unable to disclose themselves through speech. To avoid this bias, I take inspiration from Kristin Zeiler’s (2014) writings on personhood in dementia care, in which she argues that personhood is embodied and intercorporeal, that is, co-constituted through our bodily co-existence in the world. Consequently, care situations can be achieved where the personhood of the person with dementia can be expressed and upheld by means of non-verbal, bodily interaction. Zeiler’s example is joint musical activity.

Adding the dimension of embodiment and intercorporeality, we can draw the conclusion that speech is not a necessary condition for the expression of personhood. We therefore need to nuance Arendt’s account somewhat and state that non-verbal action is also revelatory. This, though, remains consistent with Arendt’s own statement that the “disclosure of who somebody is, is implicit in both his words and his deeds” (Arendt [1958] 2018, 178). Speech might be good at revealing who someone is, but non-verbal action is also capable of it, and the musical activity in Zeiler’s example is an example of action, in Arendt’s sense of the term, since the participants’ deeds cannot be prescribed beforehand, but must be adapted to the persons involved and the circumstances of the situation. More will be said about this shortly. The conclusion I draw here is that person-centred care, in its ambition to disclose who the patient is, requires action, actions that often, but not always, take the form of speech.^12^

## Who are you?

As we saw earlier, listed in the renal care coordinators’ job descriptions are three essential tasks: to coordinate the professional team around a patient; to provide individualised information and education to her or him, and to have recurrent conversations with her or him. Attached to these formal tasks are a number of anticipated results: patients should become more knowledgeable about their disease; increase their ability to engage in self-care; become more involved in their care; experience a feeling of security; and become able to make decisive treatment choices. When, during my interviews with the renal care coordinators, I asked them to describe the goals of their practice, they both reiterated some of these anticipated results and went beyond them. Let us use Britta’s account to illustrate this. “My goal,” Britta says, “has always been to… really try to *meet* the patients as far as possible.” Doing so requires an “open-ended”^13^ approach, she tells me and continues:

I gladly dedicate a couple of meetings, if I have time, to feel their [the patient] pulse. Who is this person? What has their life been like? Do they want to talk about it [the disease and treatments] or not? [she pauses for a while] Well, it’s called creating an alliance. You know, trying to build some kind of trust.

Elaborating on this further, she says:

I have an assignment to fulfil. I want to do this in a respectful manner. […] And I want to bring the person in question along. [I want them] to believe in themselves more, so that they themselves understand how much influence they have, because they indeed have. And for them to believe in themselves and agree to do these things, engage in self-care for example, I have to build trust. If I don’t, it won’t work.

In addition, Britta also emphasises the importance of helping patients understand what they have been afflicted by. Not infrequently, it takes a while before patients realise how sick they are, and what this entails. While such a realisation may provide the ground for a deeper conversation about what can be done and how one can live with the disease and its treatments; it may also give rise to a profound feeling of insecurity. Therefore, one of Britta’s main goals is also to help patients experience a feeling of security.

As we can see here, Britta reiterates some of the anticipated results found in her job description. Among her goals are to increase patients’ knowledge about their disease, help them experience a sense of security, and motivate them to engage in some form of self-care. However, she also goes beyond the formal goals of her practice, emphasising the importance, in her view, of trying to truly meet the patient, of being open, of getting to know the person, of building trust, and of being respectful, all of which are aspects of person-centred care that we recognise from the literature (see e.g. Beach et al. 2005; Kitson 2018; Toro and Martiny 2020).

Three central and mutually dependent aspects of her work not found in her job description emerge from these accounts. First, unlike the formal objectives, where the focus is on transformations of the patient resulting from the renal care coordinators’ fulfilment of their tasks, Britta’s own goals are oriented towards her practice, towards her conversations with patients and the relationships established through them. Second, in order to ensure that she is open-ended, Britta has to turn her gaze towards herself and thematise her own actions in relation to the patients she meets. Finally, her work involves trying to get to know *who* the person she meets is, getting a sense of this person’s past and how they understand and feel about their present and future. All three of these aspects are mutually dependent. Getting to know the person in front of her will not be possible if she is not open to them and focused on creating a conversation and relationship that builds trust and where they truly meet, an I-Thou meeting, to use the vocabulary so common in research on person-centred care (see e.g. Kitwood 2019; Tieu et al. 2022). But without a willingness to get to know the other person she will neither be open towards their individuality nor be able to accomplish a true meeting between them.

What furthermore unites these three aspects of renal care coordination is that they require speech and action, in Arendt’s sense of these terms. What Britta aims at in her practice cannot be conceptualised in terms of work, since her goals can only be fully understood in light of the particularities characterising each meeting with a patient, factors unique to each conversation. Characteristic of work, in Arendt’s view, is that the intended use and meaning of the end product is independent of the activities required to realise it. For Britta, aims and actions are inseparable, because what amounts to a good conversation and relationship, in her view, depends on what she determines to be the right thing to do and say in each situation, in relation to each person she meets.^14^ As Arendt puts it, “the end (*telos*) is not pursued but lies in the activity itself” (Arendt [1958] 2018, 206–207; see also Loidolt 2017, 200). Consequently, the character of the conversations and relationships Britta has depends on *who* is revealed through them. What is initiated in each meeting, then, is something qualitatively new, which can only be the result of action.

## Unpredictability, uncertainty, and irreversibility

As Arendt shows us, it is the newness and worldliness of action that makes it unpredictable, uncertain, and irreversible. In my interview with Ingela, this character of action emerges with striking clarity. When she was new in her role as renal care coordinator, Ingela tells me, she would consult detailed checklists that told her what to say and do during her meetings patients. As she became more experienced, however, she put the checklists aside and told herself: “I’m just going to walk into this.” By then, she says, she knew the theory behind her practice, so she felt comfortable not preparing herself as she used to. “I have the direction I want to go in the back of my mind,” she told herself, “but today I don’t know what’s going to happen, because it depends on who I have in front of me.”

Here we do not only see the inherent unpredictability and uncertainty of action, but also their close connection to the disclosure of who someone is. As Arendt tells us, in order to disclose who someone is, we must act—either in the form of speech or non-verbal action. All of the coordinators tell me that if they did not try to disclose who the person in front of them is, thereby letting the unpredictable and uncertain into the practice, they would not be able to help this person come up with an answer to the question why and how they should live with chronic kidney disease. This is a question without a general answer; it must be answered by and from the point of view of a particular person. This is complicated by the fact that “when you understand that you have a chronic disease, and that you’re going to end up in one of these treatments, you lose your footing, you lose your confidence,” Ingela says. “You become a ‘chronic’ [a chronically ill person],” she continues, “a burden… And all this grief over a body that no longer obeys and over what the consequences of this will be.” What Ingela says here is that the experience of being diagnosed with a chronic disease can cause a loss of one’s sense of who one is. Without such a sense, she tells me, taking one’s medication, altering one’s diet, preparing for a life with life-sustaining treatments, or even continuing to live on at all can appear meaningless. One of Ingela’s main tasks therefore is to help the patient regain a sense of who they are, by encouraging them to tell her about themselves, but also by helping them understand that they to a large extent remain the person they were before they fell ill, by saying: “You’re still a mother, wife, and grandmother […]. You’re still a teacher and flight attendant […]. You’re not only a chronic disease. It’s actually only a small part of you. You are still you, you know.”^15^

As a healthcare professional, then, one cannot simply assume that a “structured narrative” can be “obtained” from the patient, as some literature on person-centred care presupposes (Fors et al. 2016, 188; Ekman et al. 2012, 1114). According to Arendt, the disclosure of *who* someone is, cannot be achieved by one person alone. It relies on speech or other forms of action, which are “entirely dependent upon the constant presence of others” (Arendt [1958] 2018, 23). Consequently, the disclosure of who a sick person is and why and how she or he should live with the disease must be the result of a common endeavour. It must be relational, as previous research on person-centred care has already noted (see e.g. Kitson 2018; Clare et al. 2020). However, what Arendt helps us understand is that the relations that are formed in this form of care, rather than reducing the unpredictability and uncertainty characterising the sick person’s situation, create an opportunity to face it, explore it, and find new avenues of action in it, actions that do not put an end to but initiate new processes of unpredictability and uncertainty. I concur with Kristensson Uggla, who acknowledges the unpredictability and uncertainty characterising clinical situations by stating that Ricoeur’s little ethics boils down to a person’s capability of making “wise judgements in unpredictable situations characterized by great uncertainty” (Kristensson Uggla 2022, 5). However, as my analysis of the interaction between the patient Fredrik and the renal care coordinator Pia below will show, person-centred care is not only about taming unpredictability and uncertainty through wise judgement, but also about opening up for them by engaging in joint action.

Let me begin by quoting a rather lengthy excerpt from the fieldnotes and audio recording that I made during my observation of Fredrik’s and Pia’s meeting.

Fredrik is a man in his fifties. Pia and I meet him in the corridor outside his doctor’s consulting room. Together we climb the stairs to get to the floor where Pia works. Here she has furnished a special room for her conversations with patients. The room is not cosy, but nor is it conspicuously medical. It is clearly intended for conversations. In the far end, by the windows, there are three armchairs facing each other and a small table in the middle. Fredrik and Pia settle down, facing each other, and I try to place myself so I can see them both.

Fredrik and Pia have known each other for three years by now and have met on several occasions. But it has been six months since their last meeting. Pia begins the conversation by asking what the doctor, whom Fredrik has just met, said. Fredrik summarises the doctor’s words by saying: “Well… the function has decreased a little,” referring to the state of his kidneys. This compels Pia to ask how this makes him think. “When the doctors says, ‘this is how it is’, what do you think then?,” she asks. “Well, I don’t think that much,” Fredrik replies, “the only thing I think about is that I’ve been very, very flat for a long time, and now the curves are turning upwards a little. I have to shape up.” Fredrik continues by telling Pia that for as long has his curves have remained flat, he has been “cheating,” drinking juice directly from the box and eating too much protein even though he knows that it is not good for him. “I live a quite hard life, you know, in terms of work,” he tells Pia, “so then I also have to manage the other part, because I know that my work wears at my body all the time too.”

A bit further into the conversation Fredrik and Pia discuss his medications and the various symptoms to which renal care can give rise. “For me it’s all so hidden still,” Fredrik says, referring to the fact that he is yet to experience any disabling symptoms of the disease. Still, he tells Pia, his doctor said that she wants to start arranging meetings with his relatives to discuss the prospect of organ donation, that is, ask if any of them would be willing to donate one of their kidneys to Fredrik when that day comes. But Fredrik had told the doctor that he needs more time. At the moment, arranging such meetings “would be difficult,” he says, “because I’m quite alone in this.” “*I’m* prepared for it, that it can [get worse] quickly,” he continues. “It’s going to come more as shock to my family really. […] So I have to tell them repeatedly: ‘I’m sick, I’m sick, I’m sick’. And then perhaps they start thinking: ‘Maybe he’s sick’.” “We touched upon this last time we talked,” Pia replies, “exactly what you’re telling me now, that you feel that it’s difficult to put this message across to your loved ones.” “Yes, we discussed it then and since then I’ve thought it through,” Fredrik says, “and considering all I’ve been through as a person, it’s very difficult for me… to even bring up the issue. I almost need one of my relatives to volunteer to donate. I will never ask [his voice cracks up a little].” “No,” Pia replies and pauses before she continues: “But how should we solve this then? How shall we come up with [a plan]? Do you have any sort of plan already or…?” But Fredrik does not, which promotes Pia to initiate further explorations into this difficult issue. She asks: “What do you think will happen when you tell them [his relatives] that [you will eventually need a kidney donation]… ?” “Well, I don’t know,” Fredrik replies, “I don’t have that close contact with them, so it’s very difficult to know.” He tells Pia that his older sister is only his half-sister and that his younger sister has recently fought herself back to work from several years on sick leave. “I don’t even know if she would make it,” Fredrik says. “No,” Pia replies, “it’s a very delicate question. On the one hand, it depends on the relation one has to one’s loved ones. On the other hand, it depends on how they receive it. Because, as you said, she might feel… [Pia pauses] well, it’s so difficult to know how one should…”

A bit later, Pia initiates a change in focus. She says: “If we think… that it’s going to be difficult to find someone close to you who would be able to donate a kidney. What are your thoughts on that? If we change the scenario….” “Well, that would be the least problematic option for me,” Fredrik replies. “How do you mean?”, Pia asks. “Because I accept it the way it is,” he says and continues: “It’s probably the one’s closest to me that won’t accept it.” “Ok, so that would still be the case, that they would have difficulties with it? Because if one does not find a donated kidney, and you get transplanted, you’ll end up in a situation where you’re in need of dialysis,” Pia says. “In fact,” Fredrik replies, “I think that me starting dialysis would be the best way to make them reconsider their way of thinking.” “As long as I’m not on dialysis, they don’t see the problem. […] They ask: ‘How are you doing?’ [And I reply] ‘Well, as usual. Working’.” Fredrik then goes on to tell Pia that he is a trained surgical nurse, and that this training has all along afforded him another perspective on his disease than that of his closest kin. He also tells her that he has suffered from severe periods of depression, during which he has considered taking his life. “So, I’m not afraid to die,” he concludes. To ask one of his loved ones for an organ donation “would be ten times more difficult,” he says. Attempting to relieve some of the weight off Fredrik shoulders, Pia replies: “There is a resistance in the situation, to put it that way. And it’s important for you not to go through fire and expose yourself to things you don’t want. […] Do you understand what I mean? If you don’t want to ask, you shouldn’t, because it can initiate….”

Pia does not finish the sentence. Still, she has clearly alluded to the unpredictability and uncertainty characterising Fredrik’s situation and the courses of action that they explore together.

From the outset of their conversation, it is obvious that Fredrik and Pia already know each other quite well. To a certain extent, therefore, Pia knows what she can expect from their meeting. They do not have to start from the beginning, but can pick up where they left off the last time they met. Yet, their conversation is characterised by a great degree of unpredictability and uncertainty. Even though she has a sense of who Fredrik is, she does not know today, as Ingela expressed it above. The conditions under which human beings live are variable, the fact of which chronically ill persons are often painfully aware (Gunnarson 2016, 162). They must always be ready to either encounter or initiate the new. Fredrik, for example, has learnt that his curves are turning upwards, that his kidney function is decreasing. In order to help him deal with this, Pia has to understand what this means for him, which is why she begins their conversation by asking: “When the doctors says, ‘this is how it is’, what do you think then?” In asking this question, she is not interested in what is possible to think about this in any general sense—which she is probably more aware of than Fredrik—but what the particular person in front of her, Fredrik in this case, thinks about it. Thus, asking this question means continuing to ask the question “who are you?”, which entails opening up for the unpredictable and uncertain.^16^ As such, Pia’s conversations with patients take the form of continuous explorations (cf. Toro and Martiny 2020). She cannot view her goals as unrelated to the steadily altering circumstances of the person she has in front of her. Rather, she constantly has to reformulate the former in light of the latter. She must act, that is, engage in actions which, in her case, most often take the form of speech expressed as open-ended questions aiming at revealing new dimensions of the person she faces and the issue they face together. In doing so, Pia creates a space of appearance that enables Fredrik to speak and act in a way that reveals *who* he is, which, in turn, allows them to engage in a joint exploration of novel courses of action, based on the unique circumstances of the situation and their relation. What is created is a form or relational autonomy, or freedom in Arendt’s sense of the term, which takes the form of speech and action. That this is a joint endeavour, a form of cooperation, is often alluded to by Pia in the conversation, for example when she asks Fredrik: “How should we solve this then? How should we come up with [a plan]?” The issue they face is their common problem, not something Fredrik can or should deal with on his own. The search for a plan to deal with this problem is inextricably bound up with an equally common and continuous search for an answer to the question who Fredrik is in all this.

Moreover, the very situation Fredrik is in and the life-altering choice he faces together with Pia constitute another source of unpredictability and uncertainty. Fredrik and Pia dedicate much of their conversation to an exploration of what it would mean for him to bring up or not bring up the issue of organ donation with his loved ones. Clearly, Fredrik’s whole world is drawn into this choice. What he decides to do will alter his familial relations, his working life, as well as his opportunities in general. This is certain. But what courses of action there are and what consequences these will have are highly uncertain. Both Fredrik and Pia express this uncertainty during their conversation. Pia is not a neutral pillar on which Fredrik can lean while he himself decides what to do. On the contrary, they share the uncertainty and the difficulty of deciding what is the best course of action. They are both aware of the fact that the actions they choose will initiate irreversible processes that will involve numerous others and thereby quickly go beyond their control.

## Person-centred care and evidence-based medicine

This can be contrasted to the pursuit of certainty that characterises contemporary medicine. The ideal of “medical certainty,” which is rooted in the scientific approach of modern medicine, has been further reinforced since the 1990s and the entry of evidence-based medicine (Matthews 1995; Nord 2002). In evidence-based medicine, certainty is sought by founding clinical decisions on “the best available external evidence together with individual clinical expertise” (Salloch 2017, 61). In recent years, this approach to medicine has come under considerable criticism, among other things for overestimating the value of evidence from randomised control trials and underestimating other. Yet, the pursuit of certainty, in some form, is generally accepted (see e.g. Nord 2002). For good reasons. This pursuit has been, and still is, instrumental for the development of the therapeutic capability of contemporary medicine.

But how does person-centred care fit into the picture? As we have seen above, person-centred care initiates processes that are inherently unpredictable, uncertain, and irreversible. The question is, can such processes co-exist with the pursuit of medical certainty? Judging from my empirical material, it is not always uncomplicated.

Contrary to evidence-based medicine, in which certainty is sought in a cumulative process where scientific evidence, clinical expertise, and the results of various tests and examinations are added together to result in a decision about what to do, more evidence and information about various clinical alternatives does not necessarily move the process forward in person-centred care. The practice cannot be subjected to such cumulative linearity.

Fredrik’s doctor wants to establish whether someone in his family is willing and suitable for donating a kidney to him. Fredrik, however, does not feel ready to initiate such a process, and doubts whether he ever will. In this situation, Pia would not solve Fredrik’s dilemma by providing him with the latest scientific evidence about the benefits of living kidney donation. Judging from their conversation, Fredrik is already quite well versed in these matters. The dilemma he faces extends beyond the medical realm while being inseparable from it. Fredrik’s knowledge about his medical condition and the treatments available to him, is good to have, if not necessary—it helps him grasp an essential dimension of his situation. However, it remains insufficient. In order to get to the bottom with his dilemma, Fredrik and Pia must engage in an open-ended, unpredictable, and joint exploration into the particularities of Fredrik’s situation and the ways it is related to the medical alternatives. Since this requires speech and other forms of action, it is not something Fredrik can do in isolation. He needs another—in this case Pia—for whom he can appear as someone. At the end of their conversation, they agree on some things to do. Yet, the direction in which their decisions will take them is still highly uncertain. What they decide to do and say will initiate new and irreversible processes. Whether they will reach the level of certainty requested by Fredrik’s doctor is itself difficult to predict.

This co-existence of certainty and uncertainty can sometimes complicate the cooperation between renal care coordinators and doctors. From the medical perspective a certain temporal duration should result in the cumulation of sufficient evidence for making a decision. But this is not always the case in person-centred care. “There are still many [doctors],” Britta tells me, “who think that our role is only to provide information from me to you, nothing else.” “And then they breathe down your neck, not exactly thinking that you’re doing a bad job but [saying]: ‘why hasn’t this patient made a decision about which treatment they want?’ ‘Well, [Britta replies], because we have met five times now and all the patient does is cry over a lot of things. I can’t get through on the line if I don’t try to create an alliance with the patient and follow them. That’s why it’s taking time’.” What Britta would prefer is that the doctors, to a greater extent, felt a responsibility to seek up the renal care coordinators with the proposal to initiate a dialogue about the patients they share. Implicit in Britta’s words is that this would require an awareness and understanding among the doctors of the need of the kind of open-ended explorations together with patients that she and her colleagues engage in. Kristensson Uggla has something similar in mind. In his view, Ricoeur’s theory of personhood offers a way of transcending the opposition between what he terms “naturalistic medicine” and “phenomenological caring,” since it integrates the perspectives of the first, second, and third person (Kristensson Uggla 2022, 2). However, his analysis remains at a theoretical level, and does not discuss the nature of the opposition it identifies. Therefore, it does not acknowledge the problem of the co-existence of certainty and uncertainty in clinical practice that I have identified here.

## The inherent unpredictability, uncertainty, and irreversibility in person-centred care

In this article I have argued that person-centred care entails asking the question “*Who* are you?”. In other words, it involves an ambition to disclose who the health-seeking person is. With the help of the philosophy of Hannah Arendt, and through the example of renal care coordination, I have shown that such disclosure requires speech and/or other forms of action. These are modes of human activity, according to Arendt, that begin something new and insert someone new into the world, and which therefore carry with them unpredictability, uncertainty, and irreversibility.

As my empirical explorations have shown, such unpredictability, uncertainty, and irreversibility characterise both the form and content of the conversations patients and renal care coordinators engage in with each other. Thus, neither of them initiate or face these implications of person-centred care alone, but rather in and through a dynamic and explorative cooperation, in which the caregiver is not a neutral pillar on whom the patient can lean while making her or his decisions, but an involved partner that shares and has a share in the unpredictability, uncertainty, and irreversibility.

These are implications of person-centred care that research on the subject has to date not fully grasped. Person-centred care can neither be standardised as repeatable models and evaluated through randomized control trials, nor can its unpredictability, uncertainty, and irreversibility be fully tamed by professional judgement. The latter three aspects are inherent in it, since it relies on action and is oriented towards getting to know *who* the patient is. If medicine is to be person-centred, then, it has to find a way to accommodate these aspects. The question is if and how this is possible, considering its aim to establish certainty.

The limited space of this article does not allow me to give a full answer to this question. My ambition here is merely to acknowledge the tension between these overlooked yet central implications of person-centred care and the main direction taken by contemporary medicine. Perhaps the beginnings of an answer to this question can be found in Britta’s wish for continuous dialogue not only with patients but also with doctors or in Ricoeur’s theory of personhood, as suggested by Kristensson Uggla. A fuller answer to this question, however, requires further philosophical and empirical exploration. What also needs further consideration is the role and character of the professionals’ personhood, which this article has merely alluded to as a central aspect of person-centred care.

## Footnotes

1 In such a relation both patient and caregiver emerge as persons. Several scholars have argued that person-centred care should acknowledge and care for the personhood of all involved actors, be it professionals, patients, or relatives (e.g. Buetow 2016; Kadri et al. 2018). My focus in this article, however, is primarily on the ways in which the interaction between a single caregiver and a single patient discloses *who* the patient is and what the implications of this disclosure are for the actors involved as well as for clinical practice in general. As I will try to argue, since it must always pose the question “who are you?”, the implications I identify are relevant for person-centred care in all its configurations.

2 My ambition here is to give an example of the application of Ricoeur’s theory of personhood in the context of person-centred care, not to offer an exhaustive account of this theory.

3 For two other applications of Ricoeur’s “little ethics” on person-centred care see Ekman (2022) and Jobe (2022).

4 The model consists of three consecutive “routines”: “initiating the partnership,” in which the caregiver invites the patient to tell his or her “patient narrative”; “working the partnership,” in which a process of “shared decision making” commences, based on a joint deliberation between the two partners; and “safeguarding the partnership,” in which the communication between and the actions taken by the two partners are documented (Ekman et al 2011, 250).

5 The title of the project was *The Role of Existential Philosophy in Health Care: The Cases of Germany, Denmark, and Sweden* and it was funded by the Baltic Sea Foundation.

6 The fourth coordinator had then left her work as a renal care coordinator, and was thus unavailable for an interview.

7 Since I am not a trained renal nurse, I would neither be allowed nor able to actively participate in their work.

8 If the opportunity to obtain an oral consent from the patient to audio record the conversation did not arise before it began, I did not turn on the recorder. During these conversations I took notes in a small notepad.

9 Some of the questions I had prepared were: “How would you describe your role?”; “What is it like to engage in extended conversations with patients?”; “After a while, it seems like you get to know the patients a little, what does this mean for your relation, your conversations, and your work?”; “How would you describe your role in relation to the choices that patients face?”.

10 This analysis also gave rise to other central themes, which will hopefully be dealt with in future publications. For example, “the moral dimensions of self-care,” which concerns the ways in which the moral charge of many of the self-care practices that were discussed in the meetings emerged and were dealt with by patients and coordinators, “the personhood of the caregiver,” which concerns how the personhood of the coordinators was disclosed in the meetings and how they reflected about this in the interviews, and “knowing disease rather than experiencing illness,” which concerns how the fact that most patients got to know that they suffer from a chronic disease before they experienced any illness symptoms affected the interaction between patients and coordinators.

11 Ref. No. 2018/922–31/2.

12 This conclusion is supported by recent phenomenological interpretations of Arendt in which speech is understood as a form of action and action is seen as embodied (see Loidolt 2017; des Portes 2021; Hyvönen 2021).

13 She uses the Swedish word “förutsättningslös,” which lacks a clear equivalent in English. It can also mean “unconditional” or “unbiased”.

14 As I argued above, this is the case also in non-verbal person-centred care, as in Zeiler’s (2014) example of joint musical activity in dementia care.

15 This echoes research on person-centred care conducted in the context of dementia, where this form of care has been found to promote a continuation of a sense of self (Edvardsson et al. 2010; Clare et al. 2020; Tieu et al. 2022).

16 Remember that, according to Arendt, speech is revelatory as long as it concerns something of mutual interest (Arendt [1958] 2018, 182).
